# Psychological responses and factors associated with depression and anxiety in entry personnel under quarantine during pandemic in China

**DOI:** 10.3389/fpubh.2024.1368463

**Published:** 2024-10-03

**Authors:** Liping Chen, Qiao Chu, Chenhui Xu, Feng Zhou, Xiaolong Hu, Zhaoxin Wang, Ying Jin, Yipeng Lv

**Affiliations:** ^1^Huangpu District Dapuqiao Community Health Center, Shanghai, China; ^2^School of Public Health, Shanghai Jiao Tong University School of Medicine, Shanghai, China; ^3^Department of Orthopaedics, Shanghai Changzheng Hospital, Second Affiliated Hospital of Naval Medical University, Shanghai, China; ^4^Huangpu District Center for Disease Control and Prevention, Shanghai, China; ^5^The First Affiliated Hospital, Hainan Medical University, Haikou, China; ^6^Key Laboratory of Urban Complex Risk Control and Resilience Governance, Shanghai Emergency Management, Shanghai Jiao Tong University, Shanghai, China

**Keywords:** quarantine, anxiety, depression, stress coping, pandemic

## Abstract

**Background:**

The global COVID-19 pandemic has highlighted critical concerns surrounding mental health. Social isolation measures, such as the quarantine of incoming travelers, are essential public health strategies for the prevention and control of infectious diseases. However, quarantine can lead to adverse psychological outcomes, including feelings of confinement, boredom, perceived scarcity of supplies and information, financial hardship, and social stigma. This study aims to assess the mental states of quarantined individuals, investigate the factors affecting their mental well-being, and examine their coping mechanisms, with the objective of providing recommendations to enhance mental health in anticipation of future outbreaks, such as Disease X.

**Methods:**

We surveyed 327 individuals in quarantine from September 22, 2020 to January 9, 2021, collecting general demographic data and information related to COVID-19. Depression and anxiety were assessed using the PHQ-9 and GAD-7 scales, respectively, while stress coping was evaluated with a simplified version of the Cope scale. We analyzed the relationships between independent variables and mental health outcomes.

**Results:**

Among the individuals undergoing entry quarantine, 27.8% reported symptoms of depression, and 20.5% reported symptoms of anxiety. Students were more likely to experience depression compared to those with permanent jobs or no occupation. Significant risk factors for both depression and anxiety included pre-existing health conditions, lack of medical insurance, concerns about shortages of daily necessities during quarantine, and high scores for “guilt and self-blame.” Additionally, participants who worried about the impact of the epidemic on their studies or work, and those with high scores for “denial,” were more likely to exhibit depressive symptoms. On the other hand, participants who were concerned about potential rejection or discrimination from the outside world after quarantine were more prone to anxiety symptoms.

**Conclusion:**

Attention should be paid to the negative psychological reactions of the entry quarantined personnel, especially those with pre-existing health conditions, those without medical insurance, and students studying abroad. Accurate and effective epidemic dynamic information and preventive and control measures can be provided to the public to prevent fear and stigma against quarantined personnel.

## Introduction

Infectious diseases continue to pose a significant and ongoing threat to global health. For example, the recent COVID-19 pandemic has led to over 760 million confirmed cases and 6.9 million deaths globally since its emergence in December 2019, although the actual figures are likely higher (1). Mental health is among the most significant adverse outcomes of infectious diseases and the public health measures implemented to control them. A systematic review estimated that the COVID-19 pandemic resulted in an additional 53.2 million cases of major depressive disorder globally, representing a 27.6% increase, and 76.2 million additional cases of anxiety disorders, a 25.6% rise, between January 1, 2020 and January 29, 2021. In total, major depressive disorder accounted for 49.4 million disability-adjusted life years (DALYs), while anxiety disorders contributed 44.5 million DALYs globally in 2020 (2).

Although WHO has declared that the COVID-19 no longer constitutes a Public Health Emergency of International Concern (PHEIC), this does not imply that the virus is no longer a global health threat, given the ongoing uncertainties surrounding the potential evolution of SARS-CoV-2 (3). Additionally, numerous viruses and bacteria have the potential to infect humans. WHO uses the term “Disease X” to acknowledge the possibility that a future severe international epidemic could be caused by a pathogen that is currently unknown (4). On February 12, 2024, at the World Government Summit, WHO Director-General Tedros Adhanom Ghebreyesus remarked that COVID-19 exemplified a “Disease X” and cautioned that we are likely to face another pandemic within our lifetimes. He emphasized that if such a pandemic were to occur tomorrow, we would likely encounter many of the same challenges faced during the COVID-19 crisis (5).

Quarantine is a crucial public health measure for controlling the spread of infectious diseases; however, it often entails separation from family and friends, loss of freedom, and uncertainties regarding the disease and one’s health status. These factors can adversely affect the emotional and mental well-being of individuals in quarantine. During previous infectious disease outbreaks, reports indicated a range of mental health symptoms related to quarantine measures, including intense anger, depression, fear, sadness, and anxiety. Depression and anxiety during the COVID-19 pandemic have significantly contributed to the global health burden and are expected to have long-term economic and social consequences (2).

Risk cognition refers to an individual’s subjective perception of the potential or actual outcomes associated with various risk factors, and serves as a primary internal motivator for taking specific actions. A study conducted across 112 countries found that perceptions of risk related to COVID-19 were linked to emotional responses and, ultimately, to mental health outcomes (6). Other studies have shown that a higher perceived severity of the COVID-19 pandemic is associated with more severe symptoms of depression, anxiety, and stress in individuals (7, 8). Coping strategies can significantly influence both the nature and impact of psychological responses in stressful situations and can have either protective or detrimental effects on mental health (9). Research indicated that positive coping strategies can mitigate negative emotions, while negative coping strategies were associated with increased risk of negative emotions (10). Additionally, coping strategies were significant predictors of mental health outcomes (11). Given the link between emotions, disease perception, and coping strategies, it is crucial to identify factors that influence mental health and enhance protective coping mechanisms, which could serve as preventive measures in future crises.

Although existing studies have explored the associations between quarantine and mental health during the COVID-19 pandemic (12–14), there is a gap in evidence specifically regarding individuals who undergo a 14-day quarantine immediately upon arrival in mainland China. Therefore, this study aims to investigate the mental health status of individuals subjected to this quarantine process and to identify relevant influencing factors. We seek to identify high-risk groups who may benefit from targeted psychological interventions and to enhance the psychological well-being of vulnerable quarantined individuals.

## Methods

### Study design, sampling method and data collection

A cross-sectional study design was employed to conduct an electronic questionnaire survey among participants at centralized quarantine medical observation sites, including Zhongxingjunting, Quanji, Heyi, and Home Inns in Huangpu District, Shanghai. The convenience sampling method was utilized from September 22, 2020 to January 9, 2021. Participation in the online survey was voluntary and anonymous. Quarantine personnel were informed about the purpose and content of the study, and data collection was conducted only after obtaining their consent. The study received approval from the Ethics Committee of the School of Public Health, Shanghai Jiao Tong University.

### Inclusion criteria

Participants were individuals who had been quarantined following entry into China during the COVID-19 epidemic. Inclusion criteria were: informed consent for participation in the study; ability to complete the questionnaire independently; age ≥ 14 years old, for those under 18, consent was also obtained from parents or legal guardians. Participants were excluded if they were: suspected cases or close contacts of COVID-19; individuals with cognitive impairments, mental disorders, or serious physical illnesses that prevented them from completing the questionnaire.

### Survey instrument

Based on a literature review and consultation with psychologists, our custom-designed questionnaire was consisted of five parts: I. Sociodemographic characteristics data, including nationality, gender, age, education level, marital status, occupation, income, personal health status, etc. II. Personal feelings and attitudes toward COVID-19 and quarantine were assessed using self-designed questionnaire items. This section focused on concerns related to the pandemic and quarantine measures, utilizing a 4-point Likert scale with scores of 0, 1, 2, and 3 representing “not worried at all,” “a little worried,” “relatively worried,” and “very worried,” respectively. The specific survey items are detailed in the horizontal headings of Table 1. III. Depression was tested by the Patient Health Questionnaire-9 (PHQ-9) which includes 9 items (15); a score of 0–4 is interpreted as no depression, 5–9 as mild depression,10–14 as moderate depression, and ≥ 15 as severe depression. Cronbach’s *α* of PHQ-9 was 0.895 in this study. IV. anxiety was tested by the Generalized Anxiety Disorder 7-Item Scale (GAD-7) which includes 7 items (16); a score of 0–4 is interpreted as no anxiety, 5–9 as mild anxiety, 10–14 as moderate anxiety, and ≥ 15 as severe anxiety. Cronbach’s α of GAD-7 was 0.938 in this study. V. Five subscales of “face the problem, formulate strategies, denial, guilt and self-blame, and seek emotional support” of the simplified version of Carver’s Cope scale were selected to investigate individual stress coping, with a total of 10 items and a total score of 10–40 points (17). Cronbach’s *α* of the simplified version of Carver’s Cope scale was 0.817 in this study.

**Table 1:** Description of depression and anxiety symptoms of entry quarantine personnel with different levels of concern about the epidemic and quarantine.

Item	Classification	Depression symptoms			Anxiety symptoms		
		Number (percent)	Number (percent)	*p-*value	Number (percent)	Number (percent)	*p-*value
Worried about being infected during the COVID-19	Not worried at all A little worried Relatively worried Very worried	26 (86.7) 99 (73.3) 62 (66.0) 49 (72.1)	4 (13.3) 36 (26.7) 32 (34.0) 19 (27.9)	0.169	26 (86.7) 111 (82.2) 71 (75.5) 52 (76.5)	4 (13.3) 24 (17.8) 23 (24.5) 16 (23.5)	0.415
Worried about the epidemic would affect studies/work	Not worried at all A little worried Relatively worried Very worried	49 (83.1) 104 (80.6) 39 (69.6) 44 (53.0)	10 (16.9) 25 (19.4) 17 (30.4) 39 (47.0)	0.000	51 (86.4) 111 (86.0) 43 (76.8) 55 (66.3)	8 (13.6) 18 (14.0) 13 (23.2) 28 (33.7)	0.003
Worried about the epidemic would affect economic income	Not worried at all A little worried Relatively worried Very worried	60 (80.0) 91 (76.5) 56 (75.7) 29 (49.2)	15 (20.0) 28 (23.5) 18 (24.3) 30 (50.8)	0.000	64 (85.3) 99 (83.2) 60 (81.1) 37 (62.7)	11 (14.7) 20 (16.8) 14 (18.9) 22 (37.3)	0.005
Worried about being infected during the journey	not worried at all A little worried Relatively worried Very worried	37 (78.7) 133 (69.6) 45 (77.6) 21 (67.7)	10 (21.3) 58 (30.4) 13 (22.4) 10 (32.3)	0.429	40 (85.1) 153 (80.1) 45 (77.6) 22 (71.0)	7 (14.9) 38 (19.9) 13 (22.4) 67 (29.0)	0.482
Worried about being discriminated against or treated unfairly by the outside world if on a flight with a confirmed COVID-19 patient	Not worried at all a little worried Relatively worried Very worried	54 (79.4) 99 (76.7) 60 (72.3) 23 (48.9)	14 (20.6) 30 (23.3) 23 (27.7) 24 (51.1)	0.001	64 (94.1) 107 (82.9) 64 (77.1) 25 (53.2)	4 (5.9) 22 (17.1) 19 (22.9) 22 (46.8)	0.000
Worried about having similar symptoms of COVID-19 during quarantine	Not worried at all A little worried Relatively worried Very worried	121 (80.7) 84 (67.7) 17 (60.7) 14 (56.0)	29 (19.3) 40 (32.3) 11 (39.3) 11 (44.0)	0.009	130 (86.7) 97 (78.2) 16 (57.1) 17 (68.0)	20 (13.3) 27 (21.8) 12 (42.9) 8 (32.0)	0.001
Worried about lack of daily necessities during quarantine	Not worried at all A little worried Relatively worried Very worried	178 (82.8) 37 (54.4) 13 (56.5) 8 (38.1)	37 (17.2) 31 (45.6) 10 (43.5) 13 (61.9)	0.000	189 (87.9) 45 (66.2) 15 (65.2) 11 (52.4)	26 (12.1) 23 (33.8) 8 (34.8) 10 (47.6)	0.000
Worried about losing contact with family and friends during quarantine	Not worried at all A little worried Relatively worried Very worried	188 (77.7) 31 (56.4) 8 (44.4) 9 (75.0)	54 (22.3) 24 (43.6) 10 (55.6) 3 (25.0)	0.001	206 (85.1) 33 (60.0) 12 (66.7) 9 (75.0)	36 (14.9) 22 (40.0) 6 (33.3) 3 (25.0)	0.000
Worried about being rejected or discriminated by the outside world after quarantine	Not worried at all A little worried Relatively worried Very worried	161 (80.1) 58 (66.7) 10 (43.5) 7 (43.8)	40 (19.9) 29 (33.3) 13 (56.5) 9 (56.2)	0.000	177 (88.1) 63 (72.4) 13 (56.5) 7 (43.8)	24 (11.9) 24 (27.6) 10 (43.5) 9 (56.2)	0.000

### Statistical analysis

Statistical analyses were conducted using SPSS 26.0 software. The reliability of the instrument was assessed with Cronbach’s α. Descriptive statistics were used to analyze general characteristics of the participants. The Chi-square test was applied to categorical variables, while the Mann–Whitney U test was used for comparing two sets of continuous variables, and the Kruskal-Wallis test was employed for comparing three or more sets of continuous variables. The Spearman correlation coefficient was utilized to evaluate the relationships between depression, anxiety, psychological risk factors, and stress coping. Binary logistic regression analysis was conducted to identify factors associated with depression and anxiety symptoms and to calculate odds ratios (ORs) with 95% confidence intervals (CIs). Statistical significance was set at *p* < 0.05 (two-tailed).

## Results

### Participant characteristics

Of the 330 questionnaires sent, 327 valid completed ones were recovered, with an effective response rate of 99.1%. The age of 327 entry quarantine personnel who participated in the survey was 37.93 ± 13.26 years with a range of 14–75. There were 170 males (52.0%) and 157 females (48.0%). Two hundred and sixty were from Mainland China, forty eight from Hong Kong or Taiwan, five from Singapore, four from Germany, four from the United States, two from Japan, one from Canada, one from Italy, and one from the Czech Republic.

### Depression and anxiety among the entry quarantined personnel

Participants had an average PHQ-9 score of 3.54 ± 4.67 in a range of 0–27, and GAD-7 was 2.32 ± 3.87 with a range of 0–21. Depression was identified in 27.8% of participants, with 19.6% classified as having mild depression, 3.7% as moderate, and 4.6% as severe. Anxiety was identified in 20.5% of participants, with 14.4% classified as having mild anxiety, 4.3% as moderate, and 1.8% as severe. The PHQ-9 and GAD-7 scores were moderately correlated (r = 0.685, *p* < 0.001).

There were statistically significant differences in PHQ-9 scores among entry quarantine personnel of different nationalities, different ages, different occupations, and different quarantine modes (*p* < 0.05). The PHQ-9 scores of individuals from Hong Kong/Taiwan were significantly lower than mainland Chinese and foreign nationals. The PHQ-9 scores were highest in the 26–35 age group, followed by the 14–25 age group. The PHQ-9 scores of international students were significantly higher than those of the other groups. The PHQ-9 scores were higher in individuals under the “14-day centralized quarantine” than in those under the “7-day centralized quarantine +7-day home quarantine” (Table 2).

**Table 2:** Univariate analysis of depression and anxiety of entry quarantine personnel during the COVID-19 pandemic [cases (%), M (P25, P75)] *n* = 327.

Characteristics	Number (percent)	PHQ-9 Score	*p-*value	GAD-7 Score	*p-*value
**Gender**					
Male	170 (52.0)	2 (0, 5)	0.510	0 (0, 2)	0.105
Female	157 (48.0)	2 (0, 6)		0 (0, 4)	
**Nationality**					
Mainland China	260 (79.5)	2 (0, 6)	0.002	0 (0, 4)	0.029
Hong Kong/Taiwan, China	48 (14.7)	0 (0, 3)		0 (0, 1)	
Foreign nationality	19 (5.8)	1 (0, 7)		0 (0, 3)	
**Age**					
14-25 years old	69 (21.1)	2 (1, 6)	0.037	0 (0, 4)	0.325
26–35 years old	96 (29.4)	3 (0, 6)		1 (0, 4)	
36–45 years old	67 (20.5)	2 (0, 5)		0 (0, 4)	
46–55 years old	53 (16.2)	2 (0, 5.5)		0 (0, 4)	
≥56 years old	42 (12.8)	0 (0, 4)		0 (0, 1.25)	
**Marital status**					
Unmarried	134 (41.0)	2 (0, 6)	0.070	1 (0, 4)	0.182
Married	166 (50.8)	1 (0, 5)		0 (0, 3)	
divorced or widowed	27 (8.2)	2 (0, 3)		0 (0, 4)	
**Education level**					
Junior high school and below	20 (6.1)	1 (0, 3.75)	0.399	0 (0, 1.75)	0.102
High school/ Technical secondary school/Junior college	66 (20.2)	2 (0, 4)		0 (0, 2)	
Undergraduate	129 (39.4)	2 (0, 7)		0 (0, 4)	
Bachelor above	112 (34.3)	2 (0, 5.75)		1 (0, 4)	
**Occupation**					
Student studying abroad	68 (20.8)	4 (1, 8)	0.004	1.5 (0, 6)	0.045
Have a permanent job	168 (51.4)	1 (0, 4)		0 (0, 2.75)	
Self-employed or freelance	22 (6.7)	1 (0, 5)		0 (0, 2)	
No occupation	29 (8.9)	1 (0, 4)		0 (0, 1.5)	
Retired	40 (12.2)	3 (0, 6)		0 (0, 4.75)	
**Main place to work/study**					
Mainland China	176 (53.8)	2 (0, 6)	0.108	0 (0, 4)	0.735
Hong Kong, China	25 (7.6)	0 (0, 3)		0 (0, 4)	
Taiwan, China	13 (4.0)	0 (0, 3.5)		0 (0, 2)	
Abroad	113 (34.6)	2 (0, 6)		0 (0, 4)	
Annual household income					
≤100,000 RMB	71 (21.7)	1 (0, 4)	0.614	0 (0, 4)	0.684
100,000–300,000 RMB	126 (38.5)	2 (0, 6)		0 (0, 4)	
300,000–500,000 RMB	44 (13.5)	2 (0, 6)		0.5 (0, 2.75)	
≥500,000 RMB	86 (26.3)	2 (0, 5)		0.5 (0, 4)	
**Pre-existing health conditions**					
No	277 (84.7)	2 (0, 4.5)	0.051	0 (0, 3)	0.154
Yes	50 (15.3)	3.5 (0, 8)		0.5 (0, 6)	
**Resident medical insurance or commercial medical insurance**					
No	77 (23.5)	2 (0, 7.5)	0.057	0 (0, 5)	0.195
Yes	250 (76.5)	2 (0, 4.25)		0 (0, 3)	
**Family members or colleagues/friends infected with COVID-19**					
No	320 (97.9)	2 (0, 5)	0.150	0 (0, 4)	0.045
Yes	7 (2.1)	4 (2, 7)		4 (1, 6)	
**Quarantine mode**					
14-day centralized quarantine	285 (87.2)	2 (0, 6)	0.010	0 (0, 4)	0.060
7-day centralized quarantine + 7-day home quarantine	42 (12.8)	0 (0, 4)		0 (0, 1)	

RMB: Chinese yuan. The average exchange rate between USD and CNY from September 2020 to January 2021 was 6.406–6.365.

There were statistically significant differences in GAD-7 scores among entry quarantine personnel of different nationalities, different occupations, and with family members or colleagues/friends that were infected with COVID-19 (*p* < 0.05). GAD-7 scores were lower in individuals from Hong Kong/Taiwan than in mainland Chinese and foreign nationals. The GAD-7 scores were higher in international students and those whose family members or colleagues/friends were infected with COVID-19 than in other individuals (Table 2).

Table 1 summarizes the rates of depression and anxiety symptoms among quarantined personnel who were at different levels of concern about the epidemic and quarantine. Depression had a statistically significant difference according to the fear that the epidemic would affect their studies/work (*p* < 0.001) and economic income (*p* < 0.001), the fear of being discriminated against or treated unfairly by the outside world if on a flight with a confirmed COVID-19 patient (*p* = 0.001), the fear of similar symptoms of COVID-19 during quarantine (*p* = 0.009), the fear of lack of daily necessities (*p* < 0.001), the fear of losing contact with family and friends (*p* = 0.001), and the fear of being excluded or discriminated by the outside world after quarantine (*p* < 0.001) (Table 1).

Anxiety had a statistically significant difference according to the fear that the epidemic would affect their studies/work (*p* = 0.003) and economic income (*p* = 0.005), the fear of being discriminated against or treated unfairly by the outside world if on a flight with a confirmed COVID-19 patient (*p* < 0.001), the fear of similar symptoms of COVID-19 during quarantine (*p* = 0.001), the fear of lack of daily necessities (*p* < 0.001), the fear of losing contact with family and friends (*p* < 0.001), and the fear of being excluded or discriminated by the outside world after quarantine (*p* < 0.001) (Table 1).

## Correlation between depression, anxiety, and stress coping

Correlation analysis showed that PHQ-9 and GAD-7 scores of quarantined personnel were positively correlated with “denial, guilt and self-blame, and seeking emotional support” of personal stress coping (*p* < 0.01) and were negatively correlated with “facing problems and formulating strategies” (*p* < 0.05) (Table 3).

**Table 3:** Correlation analysis between depression, anxiety, and stress coping of entry quarantine personnel during the COVID-19.

	Facing problems	Formulating strategies	Denial	Guilt and self-blame	Seeking emotional support
PHQ-9 score	−0.197	−0.170	0.155	0.211	0.182
*p-*value	0.000	0.002	0.005	0.000	0.001
GAD-7 score	−0.140	−0.127	0.208	0.200	0.203
*p-*value	0.011	0.022	0.000	0.000	0.000

### Logistic regression analysis of factors influencing the psychology of the entry quarantined personnel

We conducted logistic regression analyses by incorporating independent variables identified to be significant by univariate and correlation analyses, and variables considered to affect depression or anxiety based on expert opinion and previous literature reports, including nationality, age, education level, occupation, pre-existing health conditions, medical insurance, quarantine mode, whether they are worried that the epidemic would affect studies/work or economic income, whether worried about being discriminated, whether worried about having similar symptoms of COVID-19, lack of daily necessities,losing contact with family and friends during quarantine, and stress coping: face problems, formulating strategies, denial, guilt and self-blame, and seek emotional support.

Table 4 shows the results of the logistic regression analysis of factors associated with depression symptoms of entry quarantine personnel. Compared to students, participants who had permanent jobs and had no occupations were less likely to have depression symptoms (OR = 0.352, 95% CI: 0.171–0.726, *p* = 0.010;OR = 0.239, 95%CI:0.065–0.872, *p* = 0.030).Pre-existing health conditions (OR = 4.586, 95% CI: 2.038–10.319, *p* < 0.001), without medical insurance (OR = 0.511, 95% CI:0.268–0.972,*p* = 0.041),worry about the impact of the epidemic on their studies/work (OR = 1.562, 95%CI:1.187–2.054, *p* = 0.001), worry about the lack of daily necessities during quarantine (OR = 1.999, 95% CI:1.471–2.718, *p* < 0.001), a high total score of “denial, guilt and self-blame” (OR = 1.201, 95%CI:1.016–1.420, *p* = 0.032; OR = 1.306, 95%CI:1.075–1.587, *p* = 0.007)were significant risk factors for depression symptoms (Table 4).

**Table 4:** Logistic regression of factors associated with depression symptoms of entry quarantine personnel.

Variable	Beta	Standard error	Odd ratio (95% CI^1^)	*p-*value
Occupation				
	Student studying abroad (reference)			
Have a permanent job	−1.045	0.369	0.352 (0.171,0.726)	0.005
Self-employed or freelance	−0.955	0.643	0.385 (0.109,1.356)	0.137
No occupation	−1.432	0.661	0.239 (0.065,0.872)	0.030
Retired	−0.491	0.483	0.612 (0.238,1.578)	0.310
Pre-existing health conditions	1.523	0.414	4.586 (2.038,10.319)	0.000
Resident medical insurance or commercial medical insurance	−0.672	0.328	0.511 (0.268,0.972)	0.041
Worried that the epidemic would affect studies/work	0.446	0.140	1.562 (1.1872.054,)	0.001
Worried about lack of daily necessities during quarantine	0.693	0.157	1.999 (1.471,2.718)	0.000
Denial	0.183	0.085	1.201 (1.016,1.420)	0.032
Guilt and self-blame	0.267	0.099	1.306 (1.075,1.587)	0.007

1, CI = confidence interval.

With regard to the presence of anxiety symptoms, we found that participants with pre-existing health conditions (OR = 2.236, 95% CI:1.055–4.737, *p* = 0.036), without medical insurance, (OR = 0.475, 95% CI:0.244–0.924, *p* = 0.028), worry about the lack of daily necessities during quarantine (OR = 1.634, 95% CI:1.188–2.247, *p* = 0.003), worry about being rejected or discriminated against by the outside world after quarantine (OR = 1.839, 95% CI:1.295–2.612, *p* = 0.001), and with a high score of “guilt and self-blame” (OR = 1.410, 95% CI:1.167,-1.703, *p* < 0.001) were more likely to be anxious (Table 5).

**Table 5:** Logistic regression of factors associated with anxiety symptoms of entry quarantine personnel.

Variable	Beta	standard error	odd ratio (95% CI^1^)	*p-*value
Pre-existing health conditions	0.804	0.383	2.236 (1.055, 4.737)	0.036
Resident medical insurance or commercial medical insurance	−0.745	0.340	0.475 (0.244, 0.924)	0.028
Worried about lack of daily necessities during quarantine	0.491	0.163	1.634 (1.188, 2.247)	0.003
Worried about being rejected or discriminated by the outside world after quarantine	0.609	0.179	1.839 (1.295, 2.612)	0.001
Guilt and self-blame	0.343	0.096	1.410 (1.167, 1.703)	0.000

1, CI = confidence interval.

## Discussion

In this study, we found that 27.8% of entry quarantine personnel exhibited symptoms of depression, and 20.5% experienced symptoms of anxiety. Students were particularly susceptible to depression. Key risk factors negatively impacting mental health included pre-existing health conditions, lack of medical insurance, concerns about shortages of daily necessities during quarantine, worries about the epidemic’s effects on studies or work, and fears of rejection or discrimination. Additionally, individuals with high scores for “denia” or “guilt and self-blame” were more likely to experience negative emotions.

Anxiety, stress, and depression have been widespread globally due to quarantine and social isolation during the COVID-19 pandemic. A systematic review of 19 studies involving 93,569 participants reported that during the COVID-19 epidemic, the incidence of depressive symptoms among the general population ranged from 14.6 to 48.3%, while anxiety symptoms ranged from 6.33 to 50.9% (18). Our study, which focused on individuals undergoing a closed-loop quarantine system for up to 14 days after entering mainland China, found that the incidence of depression and anxiety fell within these reported ranges. Previous research indicates that depression and anxiety symptoms worsened on average in the first two months of the pandemic (19). Given that our study was conducted between September 22, 2020, and January 9, 2021, it is possible that the prevalence of depression and anxiety among quarantined entry personnel may have been higher during the initial phase of the outbreak.

We found that quarantined individuals with pre-existing health conditions had higher scores of depression and anxiety. During the epidemic, patients with pre-existing health conditions need to simultaneously deal with existing diseases and COVID-19 (20). With limited medical resources, the medical system often gives the highest priority to people who are positive for coronavirus, while those with chronic diseases may not receive immediate treatments (21). The main challenges of quarantine for people with chronic diseases are a reduction of daily exercise and health care, and delays in routine physical examinations or laboratory examinations (22), both of which may exert marked negative impacts. In Parkinson’s disease, studies confirmed lockdown restrictions increase levels of psychological distress and impose limitations on physical activities (23). In dialysis patients, 22.4% hemodialysis patients and 13.4% peritoneal patients were classified as having moderate or severe posttraumatic stress symptoms (PTSS), which need psychological support (24). The COVID-19 lockdown caused a disruption to the continuity of care for patients with chronic obstructive pulmonary disease (COPD), with associated worry, anxiety and disappointment (25). An Australian national survey showed high rates of depression, anxiety and stress among inflammatory bowel disease (IBD) patients during the COVID-19 pandemic, even those without a prior diagnosis of depression or anxiety had high rates of significant depression (34.9%), anxiety (32.0%) and stress (29.7%) (26). Individuals with pre-existing diseases may have a greater risk of infection with the novel coronavirus than healthy people. Once infected, they may also have higher rates of severe disease, mortality, and complications, which can independently increase their psychological burdens. Therefore, centralized quarantine sites should be equipped with full-time medical staff who are trained and provided with adequate resources to comprehensively analyze the individual’s relevant disease history and treatment needs, and to conduct ongoing disease monitoring, evaluations of treatment response, and treatment adjustments as required. If the condition changes beyond the treatment capacity offered at the quarantine point, the patient should be promptly transferred to an appropriate medical center for further management.

Literature regarding medical insurance impact during the COVID-19 outbreak is sparse. Our study found that depression and anxiety scores in quarantined people who lack resident medical insurance or commercial medical insurance were higher than in those with such insurance. During the quarantine, individuals without medical insurance, especially the older adult or those with pre-existing health conditions, may have greater concerns or psychological pressures arising from their potential financial burdens, so more attention should be paid to their physical and mental health. It is also important to recognize that the COVID-19 pandemic has led to an increase in unemployment, resulting in a loss of insurance access for many individuals (27). Some studies argue that unemployment insurance or more generous government economic policies (such as higher minimum wages, greater trade union protections, and tax credits for low-income families, etc.) can alleviate the negative associations of economic downturns with population health and promote better health outcomes (28, 29).

In this study, we showed that quarantined people who were worried that the epidemic would affect their studies or work were more prone to depression. Syed et al. also found that students and the unemployed had significantly higher depression scores during COVID-19 (30). In this study, international students accounted for 20.8%, and most were college students studying abroad. Because of the epidemic, they had to return to China and continue their studies via distance education, which disrupted their normal education and academic planning. Other studies also showed that during COVID-19, study disruption leading to feelings of uncertainty about the future as a consequence of delay in students’ graduation time, lack of practical sessions and guidance, difficulty adjusting to new norms of learning, and loss of momentum, etc. (31, 32). In addition, it is difficult for most students to accept online classes after paying high fees for studying abroad. All these factors contribute to international students feeling more pressure, leading to depression and other adverse emotions. During the COVID-19 epidemic, many people switched to working at home, and some companies implemented measures such as layoffs and reduction of recruitment plans due to difficulties in resuming in-person work (33). Other studies also showed that income loss or unemployment due to the COVID-19 pandemic was associated with higher psychological distress (34, 35). As such, there is an urgency to improve the unemployment security system and increase investment in employment and entrepreneurship subsidies. We recommend the development and promotion of health initiatives aimed at alleviating the impact of COVID-19-related unemployment on mental health.

Research on SARS, the Ebola epidemic, and Middle East Respiratory Syndrome (MERS) showed that a lack of basic supplies (such as food, water, clothing, and accommodation) during quarantine contributes to feelings of depression, anxiety and anger (36–38). Furthermore, insufficient access to these basic necessities during quarantine is linked to ongoing emotional stress even 4–6 months after quarantine ends. In the early stage of the COVID-19 outbreak, the spread of the epidemic and the implementation of control measures led to weakened material production, disrupted logistics, and shortages of daily necessities, resulting in widespread panic among the population. Quarantine sites in Shanghai distribute masks, thermometers, disinfectants, and other epidemic prevention materials to the quarantined personnel, and provide three meals a day, drinking water, coffee, etc. Other daily necessities can be obtained through online shopping. Although material supplies are available, our survey results still showed that “worrying about the lack of daily necessities during quarantine” was significantly related to depression and anxiety scores, which showed the importance of daily necessities supplies to the quarantined personnel. Therefore, efforts must be made to ensure that people’s daily life needs are met during quarantine to reduce the likelihood of negative emotional sequela.

This study showed that fear of being rejected or discriminated against by the outside world after the quarantine was an independent risk factor for depression and anxiety among quarantined people. Previous research found that individuals subjected to forced isolation were more vulnerable to discrimination and exclusion (39), and among the quarantined population, those with stigma were 12 times more likely to suffer from depression than those without stigma (40). Bigya Shah et al. also reported that COVID-19-related internalized stigma is associated with anxiety and depression symptoms, prior experience of quarantine, self-blame (41). Stigma, in essence, is a response to danger where the targets are regarded as somehow immoral. Although the public has a certain understanding of COVID-19, they may attribute fault to quarantined and infected individuals, along with their close contacts, believing them to be engaged in risky behaviors. The media has a strong influence on public attitudes, dramatic and fear-mongering misinformation across media platforms were shown to contribute to stigmatization during pandemic (42, 43). Therefore, it is necessary to guide public opinion by providing accurate scientific facts and avoid promoting a state of panic about the disease and specific groups. Public health officials should convey clear information to the entire affected population in a timely and effective manner, and explain to the public the reasons for quarantine and other public health measures. In addition, affected individuals should also receive access to information and other public health measures aimed at promoting a clear self-awareness, and should be helped to not internalize the public stigma into self-stigma.

According to Meyer (44), coping strategies can be divided into adaptive strategies (including active coping, planning, using emotional support, using tool support, positive reconstruction, religion, humor, and acceptance) and maladaptive strategies (including venting, denial, substance use, self-blame, behavioral disengagement, and self-distraction). Our study found that maladaptive strategies including “denial” and “guilt and self-blame” were independent risk factors for depression and anxiety among quarantined people. “Denial” is considered to be an avoidant coping strategy and a dysfunctional response to stressful situations. Although it can temporarily alleviate stress, in the long run, it can lead to poor health and aggravate anxiety, distress, and depression (45). Other studies also showed that the use of denial and self-blame coping strategies is positively related to stress perception, depression and anxiety (46, 47). Our findings highlight the associations between positive coping behaviors and psychosocial well-being, therefore, psychological support and intervention services can be offered to the quarantined individuals to help them develop positive thinking, adopt active coping strategies, and minimize the use of negative coping mechanisms.

This study had some limitations. First, this was a cross-sectional study, which only provided a brief snapshot of the psychological responses of quarantined individuals; longitudinal studies are needed to analyze mental health trajectories and evaluate whether these depressive and anxiety responses persist after quarantine. Second, we utilized an exploratory analysis involving a convenience sample without a specific power analysis because we were uncertain as to the relevant formula and metrics to determine the optimal sample size. As such there may be some sample size bias. Finally, internet data collection is prone to selection bias, and we cannot fully explain the questionnaire to the respondents face-to-face, so there may be respondents’ understanding bias that affects the results.

Despite these limitations, this study has several notable strengths. It is, to our knowledge, the first to provide an in-depth exploration of the psychological and emotional conditions of individuals who entered quarantine immediately upon arriving in Mainland China from abroad—an area that has received relatively little attention. The results are highly representative, offering valuable insights into the mental health of this specific demographic during the COVID-19 pandemic. Additionally, our findings provide new perspectives on the relationships between quarantine-related stress, coping strategies, stigma, and psychological outcomes. This research establishes a baseline for monitoring mental health during quarantine and offers practical implications for managing mental health during the COVID-19 pandemic and future outbreaks of Disease X.

## Conclusion

The results of this study indicate that depression and anxiety among individuals in entry quarantine are associated with factors such as pre-existing health conditions, lack of medical insurance, perceptions of the epidemic and quarantine, availability of daily necessities during quarantine, stigma, and coping strategies. These findings can aid in identifying the most vulnerable groups in such situations, for whom targeted interventions and tailored social support should be provided. Measures such as ensuring the provision of adequate information, maintaining open communication channels, securing access to daily necessities, and reducing stigma can enhance psychosocial and social outcomes during outbreaks.

## Data Availability

The raw data supporting the conclusions of this article will be made available by the authors, without undue reservation.
