# Supplementary material for: Psychological responses and factors associated with depression and anxiety in entry personnel under quarantine during pandemic in China
Source: Front Public Health. 2024 Oct 3;12:1368463. doi: 10.3389/fpubh.2024.1368463 (PMC11484827; doi:10.3389/fpubh.2024.1368463)
Supplement: Supplementary file 1 [file Presentation_1.pdf]

Survey on well being of people coming to Shanghai under COVID-19 Pandemic

Informed Consent

Dear Sir/Madam,

We would like to invite you to participate in a survey on the well being of people coming to Shanghai quarantine under the COVID-19 pandemic. Should you choose to participate, please read the following carefully. If you have any questions, you can ask the surveyor. This survey has been approved by the Ethics Committee of the School of Public Health, Shanghai Jiao Tong University.

I. Background and purpose of investigation

As COVID-19 has spread around the world, Shanghai has put all inbound travelers under quarantine for 14 days starting from 0:00 on March 28, 2020. Upon Arrival in Shanghai, entry personnel will be quarantined for 14 days. Their emotional reaction and mental health during the quarantine period are of great concern. The purpose of this survey is to understand the mental health status and psychological support needs of the quarantined personnel after entry, so as to provide more targeted humanistic care, provide effective and easily accessible mental health services, and avoid excessive anxiety and psychological pressure of the quarantined personnel.

II. Survey objects

Quarantined personnel from abroad, aged ≥14 years.For adolescents under 18 years of age, consent must be obtained from their parents or legal guardians.

III. Investigation content

In this study, online questionnaire is used, which is divided into five parts: general condition questionnaire, personal feelings or concerns related to the pandemic and quarantine, health depression questionnaire, anxiety screening scale, and individual stress coping. The expected time to complete the questionnaire is 6-8 minutes.

IV. Whether there are risks or benefits

1. Participating in the survey will not affect your life, work or study in China.

2. Your participation will be helpful for us to understand the psychological status and psychological support needs of the quarantined personnel in this major global public health event, which is of great value to the effective prevention and control of the pandemic and better isolation in the future!

V. Rights and Obligations

Your participation in the survey is entirely voluntary. Should You choose not to participate in this survey, it’s totally fine.

If you do participate in this survey, please fill in the questionnaire in your best effort.

VI. Information security

This survey is conducted anonymously, and personal information will be kept highly confidential. Furthermore, the survey information will be collected by the research team and the survey data will be kept by the research staff for research purpose only. No public will have access to this information.

VII. If there are any questions or difficulties, who should be contacted?

You may access information related to this survey at any time. If you have any questions regarding the survey, you can send an email to sunflowerclp@163.com.

After reading the above informed consent form, do you voluntarily agree to participate in this survey

□Agree □disagree

1. **Your nationality:_________**

2. **Your gender**

○Male ○Female

3. **Your age:_________**

4. **Your education background**

○Junior high school and below ○High school ○Technical secondary school

○Junior college ○Bachelor's degree ○Master's degree ○Doctor's degree

5. **Your marital status**

○unmarried ○married ○divorced ○widowed

6. **Your occupation**

○student

○ professional and technical personnel (such as teachers, engineers, lawyers, journalists, writers, etc.)

○medical personnel or health related worker

○government worker

○enterprise staff or administrator

○ business personnel (including sales, finance, trade, and private business owners, etc.)

○farmer

○individual or freelancer

○no career

○retired

○others___________

7. Your main work/study location is in

○Mainland China ○Hong Kong, China ○Taiwan, China ○Abroad

Are you going abroad on official assignment

○Yes ○No

8. What is your family's annual income

○100,000 RMB and below

○100,000-200,000 RMB

○200,000-300,000 RMB

○300,000-400,000 RMB

○400,000-500,000 RMB

○500,000 RMB and above

9. Your depature country:___________departure city:___________

10. The main reason you come to China is

○go to school

○work

○business trip

○travel

○visit relatives

11. How difficult is it for you to book flight ticket

○no difficulty ○relatively difficult ○difficult ○very difficult

12. Do you have a history of pre-existing health conditions

○No

○Yes: ○Hypertension ○Diabetes ○Cerebrovascular Disease ○Hyperlipidemia ○Chronic Bronchitis ○Chronic Obstructive Pulmonary Disease ○Chronic Liver Disease ○Chronic Kidney Disease ○Tumor ○mental and psychiatric diseases ○others

13. Do you have resident medical insurance or commercial medical insurance

○Yes　○No

14. Have you ever had a family member or colleague/friend infected with a novel Coronavirus

○Yes　○No

15. Were you worried that you might be infected with a novel Coronavirus during your journey

○not worried at all　○a little worried　○relatively worried　○very worried

16. Are you worried that you or your family will be infected by a novel Coronavirus during the outbreak

○not worried at all　○a little worried　○relatively worried　○very worried

17. If you know that COVID-19 patients are confirmed on your flight, are you worried that you would suffer from discrimination or unfair treatment from outside

○not worried at all　○a little worried　○relatively worried　○very worried

18. Are you worried that your studies / work will be affected by the epidemic

○not worried at all　○a little worried　○relatively worried　○very worried

19. Are you worried that the epidemic would reduce your income or that of your family

○not worried at all　○a little worried　○relatively worried　○very worried

20. Your quarantine mode is

○14 days of centralized quarantine

○7+7 home quarantine

21. During the quarantine

Do you worry about similar symptoms of COVID-19, such as fever and cough

○not worried at all

○a little worried

○relatively worried

○very worried

Do you worry that the necessary daily living supplies are not guaranteed during quarantine

○not worried at all

○a little worried

○relatively worried

○very worried

Do you worry about not being able to keep in touch with your family and friends during quarantine

○not worried at all

○a little worried

○relatively worried

○very worried

Do you worry about being ostracized or discriminated against by the outside world after the end of quarantine?

○not worried at all

○a little worried

○relatively worried

○very worried

22. How often have the following symptoms occurred in your life in the past two weeks

Have no interest in doing anything and feel bored

○no ○a few days ○more than a week ○almost every day

Feeling low, depressed, and hopeless

○no ○a few days ○more than a week ○almost every day

Difficulty falling asleep, staying awake, or sleeping too much

○no ○a few days ○more than a week ○almost every day

Often feel very tired and lethargic

○no ○a few days ○more than a week ○almost every day

Poor appetite or eating too much

○no ○a few days ○more than a week ○almost every day

Unhappy with yourself, frustrated, and feeling like a failure

○no ○a few days ○more than a week ○almost every day

Unable to concentrate, even when reading newspapers or watching TV, memory declines

○no ○a few days ○more than a week ○almost every day

Act or speak slowly enough to attract attention, or vice versa, fidgety, irritable

○no ○a few days ○more than a week ○almost every day

Have the idea that it is better to die, or how to hurt oneself

○no ○a few days ○more than a week ○almost every day

If you find yourself with any of the above symptoms, to what extent do they affect your work, home life and relationships

○No difficulties ○some difficulties ○many difficulties ○very difficult

23. Based on the situation in the past two weeks, please answer whether there are conditions and frequency described below

Feel nervous, anxious

○no ○a few days ○more than a week ○almost every day

Cannot stop or control worrying

○no ○a few days ○more than a week ○almost every day

Worrying too much about all kinds of things

○no ○a few days ○more than a week ○almost every day

It is hard to relax

○no ○a few days ○more than a week ○almost every day

Unable to sit still because of uneasiness

○no ○a few days ○more than a week ○almost every day

Become easily annoyed or irritable

○no ○a few days ○more than a week ○almost every day

Feel as if something terrible was going to happen and fear

○no ○a few days ○more than a week ○almost every day

If you find yourself with any of the above symptoms, to what extent do they affect your work, home life and relationships

○No difficulties ○some difficulties ○many difficulties ○very difficult

24. When you encounter difficulties or stress events in your life, please answer the following questions according to your actual strategy or behavioral response

I will try my best to solve the problems I meet

○no ○rarely ○sometimes ○often

I will take action to make things better

○no ○rarely ○sometimes ○often

I will try to think of a strategy to deal with the problem

○no ○rarely ○sometimes ○often

I struggle to think about what to do next

○no ○rarely ○sometimes ○often

I keep saying to myself, "It's not true."

○no ○rarely ○sometimes ○often

I will refuse to believe that this has happened

○no ○rarely ○sometimes ○often

I will criticize myself

○no ○rarely ○sometimes ○often

I will blame myself for what has happened

○no ○rarely ○sometimes ○often

I will seek emotional support from others

○no ○rarely ○sometimes ○often

I will seek comfort and understanding from others

○no ○rarely ○sometimes ○often
